# Criteria for clinical audit of women friendly care and providers' perception in Malawi

**DOI:** 10.1186/1471-2393-8-28

**Published:** 2008-07-22

**Authors:** Eugene J Kongnyuy, Nynke van den Broek

**Affiliations:** 1 Child and Reproductive Health Group, Liverpool School of Tropical Medicine, UK

## Abstract

**Background:**

There are two dimensions of quality of maternity care, namely quality of health outcomes and quality as perceived by clients. The feasibility of using clinical audit to assess and improve the quality of maternity care as perceived by women was studied in Malawi.

**Objective:**

We sought to (a) establish standards for women friendly care and (b) explore attitudinal barriers which could impede the proper implementation of clinical audit.

**Methods:**

We used evidence from Malawi national guidelines and World Health Organisation manuals to establish local standards for women friendly care in three districts. We equally conducted a survey of health care providers to explore their attitudes towards criterion based audit.

**Results:**

The standards addressed different aspects of care given to women in maternity units, namely (i) reception, (ii) attitudes towards women, (iii) respect for culture, (iv) respect for women, (v) waiting time, (vi) enabling environment, (vii) provision of information, (viii) individualised care, (ix) provision of skilled attendance at birth and emergency obstetric care, (x) confidentiality, and (xi) proper management of patient information. The health providers in Malawi generally held a favourable attitude towards clinical audit: 100.0% (54/54) agreed that criterion based audit will improve the quality of care and 92.6% believed that clinical audit is a good educational tool. However, there are concerns that criterion based audit would create a feeling of blame among providers (35.2%), and that manager would use clinical audit to identify and punish providers who fail to meet standards (27.8%).

**Conclusion:**

Developing standards of maternity care that are acceptable to, and valued by, women requires consideration of both the research evidence and cultural values. Clinical audit is acceptable to health professionals in Malawi although there are concerns about its negative implications to the providers.

## Background

There are two broad dimensions of quality of maternity care, namely quality of health outcomes and quality as experienced by women receiving the care [1]. Both dimensions are crucial in measuring and improving the quality of care which in turn affects utilisation of maternal health services. In some settings women's experience of care may be more important than the health outcomes because childbirth is a culturally and emotionally sensitive area [2]. This is particularly true since most users of maternity care are healthy women who need only basic care, although some users will develop conditions requiring a higher level of care [3].

There are many tools used to improve the quality of care in maternity units. These include maternal death audit, criterion-based audit, training, dissemination of practice guidelines, plan-do-study-act (PDSA) cycles, tools for process description (client flow analysis, process mapping, and cause-effect analysis), tools for data collection (direct observation, exit interviews, and focus group discussion), tools for collaborative work (nominal group technique, facilitative supervision, force-field analysis, benchmarking, and SWOT analysis), and combined models involving a combination of these tools [4-11].

Criterion-based audit (also called clinical audit) is a well known approach for improving the quality of maternity care. It is strongly supported by expert opinion as well as national and international organisations including the World Health Organisation (WHO) and the National Institute of Clinical Excellence (NICE) in the United Kingdom [2,12]. NICE defines audit as: "A quality improvement process that seeks to improve patient care and outcomes through systematic review of care against explicit criteria and implementation of change. Aspects of structure, processes, and outcomes of care are selected and systematically evaluated against explicit criteria. Where indicated, changes are implemented at individual, team, or service level and further monitoring is used to confirm improvement in healthcare delivery" [12].

**Figure 1:**
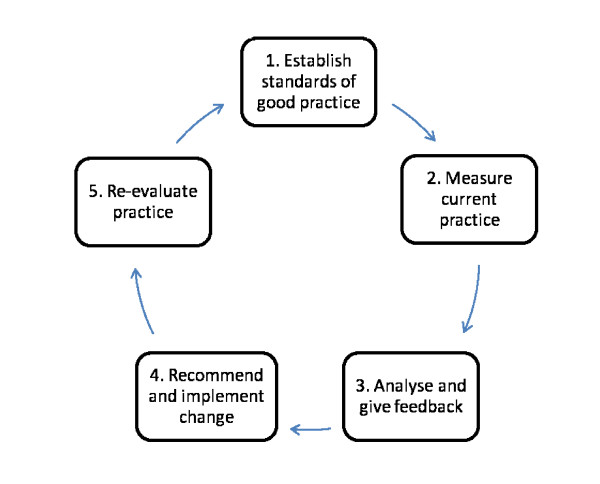
**Criterion based audit cycle.** This is a classic criterion-based clinical audit cycle with five steps. The first step is agreeing standards of good practice.

The five classic steps of the clinical audit cycle are presented in Figure 1. The first step is the development of standards. Traditionally standards have been developed by systematic reviews to identify sets of criteria that constitute optimal care. Once standards have been developed, actual practice is measured and compared with standards (best practice). Gaps in current practice are identified, recommendations are made and implemented, and progress is evaluated

Previous studies on criterion-based audit in maternity units have focused on improving the outcomes of emergency obstetric complications, ignoring quality as perceived by clients [13-15]. Criterion based audit for emergency obstetric complications has just recently been introduced in a few developing countries, including Uganda, Ghana and Jamaica [13-15]. To the best of our knowledge there are no published studies on the use of criterion based audit to improve women friendly care in a developing country setting.

Developing standards for women-friendly care is complex because (a) acceptable and valued behaviour may not be documented and (b) cultural values vary between countries or ethnic groups. Therefore systematic reviews may not capture what women consider good quality of care. This is particularly challenging in settings were traditional values strongly shape how quality of care is perceived by clients. Unfortunately this important area has been given very little attention especially in developing countries. The high maternal mortality in resource-limited settings is partly due to the poor attitudes of health providers which lead to reduced utilisation of maternity care services [16].

As part of a collaborative programme to reduce maternal mortality in Malawi, we sought to introduce criterion based audit to improve women friendly care in maternity units. This paper describes how standards for women friendly care were developed in Malawi, and attitudes of health care providers that could influence the implementation of criterion based audit.

## Methods

We developed standards for women friendly care in three districts (Salima, Kasungu, Lilongwe) in the Central Region of Malawi. The three districts have a population of 2,812,183 people with 73 health facilities (9 hospitals and 64 health centres). Standards for women friendly care were established and refined during three workshops that brought together participants from the three districts. This study was approved by the Reproductive Health Unit of the Malawi Ministry of Health.

### Establishing standards for women friendly care

#### Workshop 1: Establish standards for women friendly care

We used evidence from existing guidelines, namely Malawi national guidelines and WHO manuals [17,18]. Where necessary this was supplemented by evidence from the Cochrane database, standard textbooks and articles from peer-reviewed journals. Local standards were established during a workshop that brought together 32 participants including maternal health experts, policy makers and women's group representatives from three districts. Reproductive Health Unit of the Ministry of Health and all hospitals in the three districts were represented. The workshop was facilitated by two UK based experts who have several years of experience in sub-Saharan Africa. The participants referred to relevant publications on women friendly care throughout the standard development process. The standards were agreed by simple consensus and where there was disagreement, they refer to the WHO manuals and National guidelines. For most participants the concept of standards of care was new. The participants discussed how standards are derived and how they are 'translated' for use by stating a clear objective and working out the structure, process and outcome criteria for each objective. The participants worked through this process during facilitated (small) group work. The participants discussed lively and debated openly on subjects such as: what is feasible in our own setting, what is the Ministry of Health policy, what is current evidence based/best practice? At the end of this two day workshop, the participants agreed on eleven objectives and developed the structure, process and outcome criteria for each objective.

#### Workshop 2: Review and finalise the standards initially developed

The second workshop brought together 62 participants from health centres, hospitals, Reproductive Health Unit of the Ministry of Health and women's group representatives from the three districts. The participants reviewed the standards developed during the first workshop and agreed on a final list of criteria. During this review process, the participants considered evidence from research synthesis, relevance to the three districts and cultural values. The standards consist of 136 criteria (Table 1).

**Table 1:** Agreed criteria of standards for women friendly care

**Objectives**	**Structure criteria**	**Process criteria**	**Outcome criteria**
1. All pregnant women are received in the labour ward in a cordial manner	• staff with good interpersonal communication skills • identification badges for staff • dedicated space with a bed to receive the pregnant woman	• staff wear identification badges • clients are welcomed • clients are greeted by name. • staff introduce themselves to clients by name • staff use a language the client can understand • staff orient the woman to the labour ward/room set up	• at least 80% of women are satisfied with the reception they received at the labour ward

2. All staff have a positive attitude towards all clients	• staff with skills on interpersonal communication and counselling • staff with a positive attitude on labour ward, post natal ward, antenatal ward and clinic	• clients are welcomed • clients are greeted by name. • staff introduce themselves to clients by name • all procedures done (e.g. during labour or on the ward care) are explained by staff to the client • client is allowed to have a companion with her during labour and delivery. • clients are told how and where they can ask for assistance • patients are referred to and called by their name (and not by bed number, diagnosis etc) • staff are polite and use appropriate language	• client satisfaction of at least 80% • at least 80% of women understand the procedures undergone during labour, delivery and puerperium • at least 80% of women willing to deliver again at the at the same facility in a subsequent pregnancy • 95% of women will recommend the facility to other women • 95% of women will feel they are treated with dignity and respect

3. The cultural background of women is respected with regard to staff attitude, care during labour and delivery environment	• staff who are aware of and sensitive to the cultural background of clients • user friendly furniture especially labour bed • curtains and screens • community and clients aware that a woman is allowed and encouraged to have a companion during labour and delivery • identified companion who can be present during labour and delivery • identified space for patients to be able to interact (socialise) during hospital care	• clients' opinions are sought • staff greet clients, introduce themselves and asks about clients needs and wellbeing • labour ward environment is modified to meet the needs of the client • where possible use a low bed – if this is not possible explain why a high bed is needed and ensure client can easily climb on the bed • curtains and screens are used to help provide privacy. • clients are not exposed unnecessarily and covered with linen (unless they do not want this) • staff allow a companion to be present during labour and delivery • community and clients are informed of that having a companion during labour and delivery is allowed and of benefit. • staff identify space for clients where they can walk around, sit, socialise etc. during hospital care and encourage clients to use this. • 'Visio therapy' (e.g. information pamphlets TV or radio) are available for clients during their stay in the facility	• clients feel their cultural background is respected • clients feel they are recognised as being unique • clients and community are satisfied with care given at the facility • women delivering at the facility will feel as comfortable as at home • women delivering at the facility will have a companion wit them during labour and delivery • women will enjoy their waiting time/time at the facility learn something new • increased number of women deliver at the facility (with skilled attendant)

4. All women coming to maternity ward have the right to be treated with respect and dignity	• all staff members have good communication skills • all staff are trained in patient rights, health care provider rights and responsibilities and interpersonal communication skills. • Linen, curtains, screens	• staff receive training about patient rights, health workers rights and responsibilities and in interpersonal communication skills • staff communicate properly and are polite to clients • staff introduce themselves, call client by name, explain produces to be done, obtain informed consent, use appropriate language and allow clients to verbalise their feelings • linen, screens and curtains are used to ensure privacy: 'clients are not exposed anyhow'	• every woman attending the facility will feel they are respected, recognised as unique and treated with dignity. • women will not feel they have been exposed unnecessarily • women will be satisfied with the care they receive • deliveries at the facility (by skilled attendant) will increase.

5. Every woman should be attended to within 30 minutes of their arrival at the facility.	• volunteers, porters, drivers, guards who are aware of the need to escort clients to appropriate wards • clear directions to labour ward (and emergency ward) • direct admission to labour ward possible (i.e. not having to go to records office etc first) • skilled knowledgeable staff available 24 hours 7 days. • emergency obstetric care equipment and supplies available 24 hours 7 days.	• women in labour or with pregnancy related complications are identified and escorted to the labour ward (or relevant emergency room) at point of entry to the facility. • Support staff as well as health care providers are made aware of the need to and possibility of direct admission to the labour ward (or emergency room) for women in labour and/or with pregnancy related complications. • all staff on labour ward and emergency room commence management of patient when she arrives • protocols for management of women requiring emergency obstetric care and/or in labour are in place	• delays will be reduced • patients will feel welcome • client flow improved • clients will be attended to properly • 90% of women are assessed and initial treatment commenced within 30 minutes

6. All mothers are provided with an enabling environment.	• safe and clean environment (labour ward, emergency room) ready 24 hours 7 days. • cleaning material and disinfectant • water • agreed protocol for cleaning of labour ward/emergency room. • functioning and adequate bathrooms, toilets, washing area, guardian shelter, kitchen space • adequate number of skilled attendants	• staff keep labour room, beds, toilets, bathroom, floors, windows, walls and linen clean. • clients are provided with functioning and adequate bathrooms, toilets, washing area, guardian shelter, kitchen space	• increase in number of women attending the facility • reduced infection rates • increased client satisfaction

7. All clients to be informed of the services and treatment alternatives available at the facility	• IEC material (posters, leaflets, banners) • staff with knowledge and awareness about treatment alternatives and able to inform client in her/his own language of choices	• IEC material used • health talks on services available are given eg during antenatal clinics, via drama, via media (radio/TV and newspapers) • services explained to client by health care provider • treatment alternatives explained to client by health care provider • health care provider takes client's choice into consideration	• increased service utilization • improved provider/client relationship • clients able to make an informed choice • health care providers create a learning environment as they will themselves need to learn about treatment alternatives available. • increased working satisfaction for health care providers

8. Each client is treated according to her individual needs	• paper, pen (case notes) • staff able to take history • staff able to make management plan and implement • curtains and screens for privacy • labour room with conditions conducive for individualised delivery care	• client is addressed by name and the health care provider introduces him/herself to the client • curtains and screens are used and the client is covered with linen when examined etc. • a proper and complete history is taken for each client, including: ○ personal data (name, address, age, marital status, religion) ○ obstetric history • information obtained is actually used to decide on a management plan for the client and the management is implemented. • staff allow guardian in labour room and explain procedures • clients are allowed to adopt the delivery position of their choice (e.g. squatting, supine, kneeling) • pain relief measures are given during labour (back rubs, walking, drugs, reassurance)	• reduced number of maternal death • individual case note available • improved rapport with client • every woman attended to at the health facility feels she has been treated with dignity and respect

9. At the facility a woman receives skilled attendance at birth and emergency obstetric care when required.	• skilled staff available. • Emergency obstetric care equipment and supplies available on the labour ward and emergency room (including an emergency tray). • staff and supplies available 24 hours a day, 7 days a week.	• in-service training of staff on emergency obstetric care is conducted with regular updates. • all necessary drugs needed for delivery and emergency obstetric care are ordered in a timely manner and checks for availability are in place. • proper handovers are conducted. • patients are informed patients of services available and these are provided to them as and when necessary.	• 95% of women who come to the facility in labour or with complications requiring emergency obstetric care will have skilled attendance and emergency obstetric care provided to them as and when required. • increased uptake of skilled attendance and emergency obstetric care.

10. All client information will be treated with confidentiality and discretion	• staff that can maintain confidentiality	• staff use low tone in communication • clients opinion is sought regarding disclosure of information • clients records and condition are kept confidential (verbal and written)	• every woman attended to at the health facility feels that her information will be kept confident

11. All health workers to ensure proper management of patient information	• skilled staff on information management • data entry clerks • enough stationery (registers, papers, pens, computers, files) • lockable drawers	• orient staff on the necessity for proper recording of information: 'if it is not recorded, it did not happen' • all health care providers record all activities that are carried out for the patient. • recorded information is used (e.g. case notes referred to, used in handovers etc). • health care providers ensure that data tools are available (registry books, tally sheets, stationery, partograph, case notes etc) • all patient information is kept confidential and safe	• good data available for audit • improved information driven decision making for all women who have been seen at the health facility there is a complete and accurate set of notes. • registry books (e.g. labour ward register) accurately reflect what activity there is at the health centre

For each objective there are structure, process and outcome criteria. Each bullet point represents one criterion.

#### Workshop 3: Select criteria to audit

The third workshop brought together 60 participants. From the list 136 criteria, 14 criteria were selected to be audited within the next six months in order to assess and improve women friendly care in maternity units in the three districts.

### A survey of providers' attitudes towards criterion based audit

In order to assess attitudinal barriers that could impede the proper implementation of criterion-based audit, we conducted a survey of health care providers in the three districts (Salima, Kasungu, Lilongwe) of Malawi. We carried out this survey during a quality improvement workshop that brought together 54 participants from 9 hospitals and 18 health centres in three districts of Malawi. We collected data using an anonymous, self-administered questionnaire after verbal informed consent. Part one of the questionnaire consisted of seven "Yes or No" questions. Part two consisted of two open ended questions: first question was on the perceived challenges of implementing criterion based audit and second question was on the possible ways of overcoming the challenges. We entered and analysed the data with the Statistical Package for Social Sciences (SPSS) software programme (version 14.0, Chicago, IL, USA).

## Results

### Agreed standards for women friendly care

The standards developed for women friendly care consisted of eleven objectives, each with structure, process and outcome criteria (Table 1). The eleven objectives addressed different aspects of care given to women in maternity units: (i) reception, (ii) attitude towards women, (iii) respect for culture, (iv) respect for women, (v) waiting time, (vi) enabling environment, (vii) provision of information, (viii) individualised care, (ix) provision of skilled attendance at birth and emergency obstetric care, (x) confidentiality, and (xi) proper management of patient information.

### Criteria selected to audit in the first six months

Fourteen criteria were selected to be audited in the next six months (Table 2). The selection was based on perceived deficiencies of women friendly care in maternity units. The criteria addressed different aspects of care such as greetings, self-introduction, companionship during labour, privacy, cleanliness, provision of basic facilities (e.g. bathroom, toilets), birthing positions, communication skills, respect for women, and client satisfaction.

**Table 2:** Criteria (for women friendly care) selected to audit

1. Health worker greets all women when they arrive the health facility
2. Health worker introduces him/herself to women when they arrive the health facility
3. Health worker informs and allows all pregnant women to have a companion of their choice during labour
4. Health worker uses linens to cover women and ensure privacy during labour
5. Health worker uses curtains or screens to ensure privacy during labour and delivery
6. Health worker calls women or refer to them by their names and not by other names (e.g. bed number or diagnosis)
7. Health worker keeps the maternity ward clean (i.e. beds, floors, windows, walls, lines)
8. Health worker provides women with a clean bathroom and toilet
9. Health worker informs women of the different birthing positions (e.g. squatting, supine, kneeling)
10. Health worker allows women to adopt the birthing position of your choice (e.g. squatting, supine, kneeling)
11. Health worker speaks the language that is easy for women to understand
12. Health worker respects all women and treat them with dignity
13. At least 80% of women are satisfied with the care they receive in the health facility
14. At least 95% of women will recommend the health facility to a friend or relative

### Attitudes of health professionals towards criterion based audit

The health professionals generally held a favourable attitude towards criterion based audit: 100.0% (54/54) believe that clinical audit and feedback will improve the quality of care and 92.6% believe that audit and feedback is a good educational tool. However, there were concerns that audit and feedback would create a feeling of blame among providers (35.2%), manager would use audit to identify and punish providers who fail to meet standards (27.8%), audit and feedback could not be done routinely because it is time-consuming (13.0%), and the potential role of audit in reducing health care costs was questioned (see Table 3).

**Table 3:** Attitudes of health professionals towards criterion based audit in Malawi

***Proposition***	***Number of participants who agree with the proposition (N = 54)***	***Percentage (95% Confidence Interval)***
Criterion based audit will improve the quality of care	54	100.0 (94.6–100.0)
Criterion based audit is a good educational tool	50	92.6 (83.1–97.6)
Criterion based audit will reduce the health care costs	11	20.4 (11.2–32.6)
Criterion based audit cannot be done routinely because it is time-consuming	7	13.0 (5.9–24.0)
Criterion based audit will increase law suits against health care providers	4	7.4 (2.1–17.9)
Criterion based audit will create a feeling of blame among providers	19	35.2 (23.4–48.6)
Manager will use audit to identify and punish providers who fail to meet standards	15	27.8 (17.1–40.8)

## Discussion

This article describes the development of standards for women friendly care and the attitudes of health professionals towards criterion based audit in Malawi. A multidisciplinary team developed standards taking into consideration evidence from research synthesis and cultural values. Standards for obstetric complications address health outcomes while standards for women friendly care address women satisfaction in the care they receive [7]. Although client perception and provider perception are usually complementary, there are situations were the two perceptions can be antagonistic [18]. Optimal standards for women friendly care are those that are valued by women, and yet do not oppose evidence-based practice as perceived by health professionals. Traditionally, standards have been developed by a panel of experts and then implemented by a multidisciplinary team [13]. In obstetrics, the panel of experts is often made up of obstetricians while midwives who are usually excluded from the early stages of criterion based audit [15]. We involved all grades of health professionals (including midwives) from the very beginning of standards development process, and we hope that this will promote ownership and sustainability. We equally involved managers and policy-makers from the first step of criterion based audit, and we hope that this will facilitate the implementation of recommendations that require extra resources (e.g. finance and staff) and approval of hierarchy.

The criteria developed for women friendly care have some limitations. The list of agreed criteria is long and it might not be feasible to audit all the criteria simultaneously. Some authors have suggested that the criteria be limited to a short list that can easily be audited [4]. The participants agreed that the long list be maintained for reference, and that the criteria to be audited should be selected based on their relevance and level of priority. Alternatively the providers could audit each objective in turn until all the objectives have been audited. The participants also noted that some criteria appear under two or more objectives, e.g. greeting the client. Nevertheless they agreed that this was acceptable since the criteria address different objectives. In addition some criteria were agreed were not based on evidence (e.g. greeting) but on cultural norms and values.

## Conclusion

A survey of the health professionals in Malawi showed that they held a favourable opinion about clinical audit. However there are some areas of concerns that should not be overlooked. For instance a third of providers believe that clinical audit will create a feeling of blame among providers who fail to meet standards, and more than a quarter believe that managers will use clinical audit to identify and punish health care providers. Some challenges identified by health care providers that could hinder the implementation of standards include shortage of staff, high workload and inadequate knowledge and skills. Many of the providers surveyed suggested some possible solutions: active involvement of managers in criterion based audit, and making it crystal clear that information from audit will not be used as a basis of disciplinary sanctions.

This paper describes the first step of a clinical audit cycle: establishing standards for women friendly care. The paper also explores attitudinal barriers which could impede effective implementation of clinical audit. Developing standards is a very crucial step that lays the foundation for the remaining steps of a clinical audit cycle. A successful completion of the next steps will determine whether criterion based audit can improve women friendly care in health facilities in resource-limited countries. Our plan is to follow up and monitor the implementation of these standards using the before-and-after study design. The major challenge will be to change current practice as other authors have reported that it is easy to change knowledge and skills, but very difficult to change clinical practice [19].

## Competing interests

The authors declare that they have no competing interests.

## Authors' contributions

EJK conceived the topic, collected the data, prepared and finalised all versions of the manuscript. NvdB reviewed the manuscript for intellectual content. All authors read and approved the final manuscript. EJK is the guarantor.

## Pre-publication history

The pre-publication history for this paper can be accessed here:


